# Leaf economic strategy and stand structural complexity are associated with net woody productivity in a subtropical mixed forest

**DOI:** 10.3389/fpls.2026.1980200

**Published:** 2026-10-06

**Authors:** Juyang Wu, Jingjing Huang, Xunru Ai, Qin Xiang, Zhengxiang Wang, Jiang Zhu

**Affiliations:** 1 College of Forestry and Horticulture, Hubei Minzu University, Enshi, China; 2 Faculty of Resources and Environmental Science, Hubei University, Wuhan, China; 3 College of Forestry, Southwest Forestry University, Kunming, China

**Keywords:** community-weighted traits, net woody productivity, species relative-abundance structure, stand structural heterogeneity, subtropical mixed forest, tree size–spatial heterogeneity

## Abstract

The associations of net woody productivity with biodiversity, functional composition, and stand structure remain poorly resolved in subtropical mixed evergreen–deciduous broad-leaved forests. We used the 2016 and 2021 censuses of 150 contiguous 20 × 20 m subplots in a 6-ha permanent plot at Jinzi Mountain, central China. Net woody productivity was calculated as the annualized net change in live woody biomass. Univariate and multiple linear regressions, random forests, and piecewise structural equation modeling were used to examine relationships with community attributes measured in 2016. Live woody biomass increased from 144.78 to 167.86 t·ha^-1^, corresponding to a mean net woody productivity of 4.62 t·ha^-1^·yr^-1^. Net gains were concentrated in the tree stratum and larger diameter and height classes; classes with a diameter at breast height ≥29 cm accounted for 78.53% of community net woody productivity. In univariate regressions, productivity was positively associated with community-weighted mean (CWM) leaf thickness and negatively associated with CWM specific leaf area and leaf area. Multiple regression also identified species relative-abundance structure and stand structural heterogeneity as significant correlates. In the piecewise structural equation model, species diversity and structural diversity had significant positive direct associations with productivity. A principal component representing higher CWM specific leaf area and leaf area but lower CWM leaf thickness had a significant negative direct association. Functional and phylogenetic diversity had nonsignificant direct paths, although Rao’s quadratic entropy was negatively associated with productivity in the multiple regression. These relationships may partly reflect differences in baseline biomass stocks, which were not included as predictors. Retention of healthy large canopy trees, woody-species diversity, and heterogeneous stand structure warrants consideration in close-to-nature forest management, but its consequences for productivity require further testing.

## 1 Introduction

Forests are a major component of the terrestrial carbon sink, and maintaining their capacity to accumulate and retain live woody biomass is increasingly important as biodiversity loss and forest degradation accelerate (IPBES, 2019; Mengzhi et al., 2025; Mori et al., 2021; Pan et al., 2024). In repeated forest censuses, net woody productivity is calculated as the annualized net change in live woody biomass over a defined interval. This metric integrates biomass gains from survivor growth and recruitment with biomass losses caused by mortality (Yuan et al., 2019). It therefore describes net changes in standing live woody biomass rather than complete net primary productivity, which also includes litterfall, fine-root production, and reproductive allocation (Bashir et al., 2019; Malhi et al., 2004). Identifying the community attributes associated with net woody productivity can clarify biodiversity–ecosystem functioning relationships and inform close-to-nature forest management.

Positive relationships between tree species richness and forest productivity have been reported in forest inventories and biodiversity experiments (Huang et al., 2018; Liang et al., 2016; Paquette and Messier, 2011). However, the magnitude and direction of these relationships vary with climate, elevation, disturbance history, and stage of stand development (Fei et al., 2018; Ratcliffe et al., 2017; Zou et al., 2024). In mixed forests, interspecific differences in crown architecture, canopy position, rooting depth, and resource-acquisition strategies may enhance community-level resource capture, whereas intensified neighborhood competition and resource limitation may weaken or offset such gains (Forrester and Bauhus, 2016; Forrester and Pretzsch, 2015; Pretzsch, 2022). Species richness alone does not indicate how individuals are distributed among species, which traits are represented by common species, or how trees are arranged across size classes and space (Garland et al., 2021). Communities with similar richness can therefore differ substantially in woody biomass dynamics. Assessing these differences requires consideration of species relative-abundance structure, dominant trait composition, functional differentiation, phylogenetic diversity, and stand structural heterogeneity.

Species relative-abundance structure and community-weighted mean (CWM) traits describe complementary aspects of community composition. Diversity metrics that incorporate relative abundance distinguish communities with similar species richness but different distributions of individuals among species, whereas CWM traits summarize their abundance-weighted functional composition. The mass-ratio hypothesis links ecosystem functioning to the traits of dominant taxa, providing a basis for interpreting CWM traits (Díaz et al., 2007; Finegan et al., 2015; Grime, 1998). Variation in specific leaf area and leaf tissue investment also reflects the trade-off between resource-acquisitive and resource-conservative strategies along the leaf economics spectrum (Díaz et al., 2016; Wright et al., 2004). When absolute biomass change is concentrated in large or canopy-dominant individuals, species richness alone cannot identify which species or trait combinations contribute most strongly to plot-level productivity; species relative-abundance structure and CWM trait composition may therefore provide additional information (Gu et al., 2025; Kohyama et al., 2023). Tree-size differentiation, vertical stratification, canopy packing, and local neighborhood arrangement can influence light interception, growing-space occupation, and asymmetric competition (Ehbrecht et al., 2021; Forrester and Bauhus, 2016; Murphy et al., 2022; Ray et al., 2023; Zhai et al., 2024). Functional diversity, in contrast, represents trait dispersion among co-occurring species and is relevant to complementarity only when the selected traits capture biologically meaningful differences in resource use (Laliberté and Legendre, 2010; Loreau and Hector, 2001). Phylogenetic diversity describes the evolutionary breadth of local communities, but its association with productivity depends on whether phylogenetic relatedness corresponds to functional differences that are relevant to biomass accumulation (Cadotte et al., 2008; Paquette et al., 2015). These dimensions capture distinct aspects of biodiversity–productivity relationships (Bongers et al., 2021; Finegan et al., 2015).

Subtropical mixed evergreen–deciduous broad-leaved forests provide an appropriate system for comparing these biodiversity dimensions because evergreen and deciduous woody species coexist within stands characterized by pronounced tree-size differentiation and vertical stratification (Bai et al., 2020; Ge and Xie, 2017; Zhang et al., 2021). Previous studies in this forest type have described patterns of species composition, dominant-population dynamics, community structure, and vegetation carbon storage (Feng et al., 2018; Wu et al., 2025; Yang et al., 2021; Yao et al., 2017). Chen et al. (2025) identified community-weighted maximum tree height and wood density as important predictors of productivity in this forest type. However, whether functional and phylogenetic diversity provide additional information beyond species relative-abundance structure, leaf-trait composition, and neighborhood structural heterogeneity remains unresolved at the subplot scale.

We examined net woody productivity across 150 contiguous 20 × 20 m subplots using the 2016 and 2021 censuses of a 6-ha permanent plot at Jinzi Mountain, central China. Analyses focused on predefined baseline attributes representing multidimensional biodiversity, CWM trait composition, and stand structure in 2016. Within the community, we predicted that net woody productivity would be concentrated in the larger diameter and height classes of self-supporting woody plants (H1). Among subplots, we predicted that net woody productivity would remain associated with CWM leaf economic traits, species relative-abundance structure, and stand structural heterogeneity after accounting for the Margalef richness index (H2). We further examined whether functional and phylogenetic diversity remained associated with net woody productivity after accounting for CWM trait composition and stand structural heterogeneity.

## 2 Materials and methods

### 2.1 Study site and plot design

This study was conducted in a 6-ha permanent plot of subtropical mixed evergreen–deciduous broad-leaved forest at the Jinzi Mountain State-owned Forest Farm, Lichuan City, Hubei Province, central China (30°17′17″ N, 109°02′58″ E; **Figure 1**). The study area lies in the mountainous region of southwestern Hubei and has a humid subtropical monsoon climate, with a mean annual temperature of approximately 12.7 °C and mean annual precipitation of approximately 1450 mm (Chen et al., 2025). The natural vegetation is dominated by mixed evergreen–deciduous broad-leaved forest, in which evergreen and deciduous woody species coexist within stands characterized by pronounced tree-size differentiation and vertical stratification.

**Figure 1:**
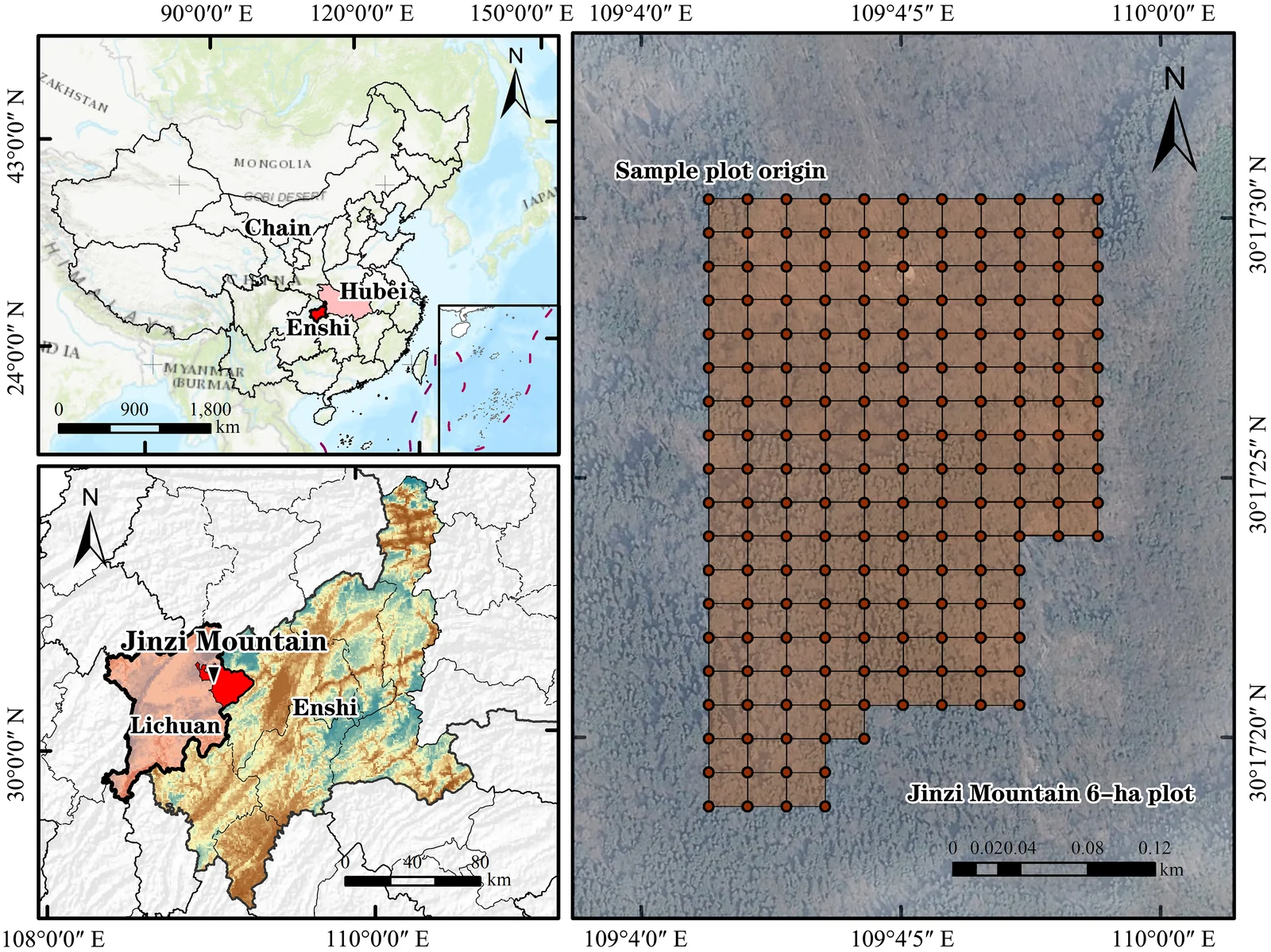
Geographical location of the study area.

The permanent plot was established in 2016 in a relatively intact natural stand with no evident recent anthropogenic disturbance. It was divided into 150 contiguous 20 m × 20 m subplots, which served as the basic spatial units for woody-plant censuses, biodiversity calculations, stand-structure analyses, and estimation of net woody productivity.

### 2.2 Forest census and estimation of net woody productivity

Complete woody-plant censuses were conducted in the 6-ha Jinzi Mountain permanent plot in 2016 and 2021. In 2016, all woody individuals with a diameter at breast height (*DBH*) ≥1 cm were uniquely tagged, identified to species, and mapped; *DBH*, tree height (*H*), and crown width (*CW*) were recorded. In 2021, surviving individuals were remeasured, mortality was recorded, and recruits reaching the *DBH* threshold were tagged, measured, and mapped. Records from both censuses were matched using individual identification numbers.

Individual live woody biomass was estimated for each census using allometric equations compiled for subtropical mixed evergreen–deciduous broad-leaved forests, with *DBH*, *H*, and *CW* used as required by the selected equation (Wu et al., 2022). Individual estimates were summed within each subplot and expressed per unit area. Net woody productivity (NWP) was calculated as the difference between subplot live woody biomass in 2021 and 2016 divided by the five-year census interval. This metric incorporates survivor growth, recruitment, and mortality losses. It does not represent complete net primary productivity, which also includes litterfall, fine-root production, and reproductive allocation (Clark et al., 2001; Malhi et al., 2004). Details of equation selection, biomass aggregation, and NWP calculation are provided in **Supplementary Methods S1.2**.

Biomass and NWP were summarized by height-defined stratum for self-supporting woody plants and separately for woody lianas. Self-supporting individuals were assigned at each census to the tree stratum (*H* ≥10 m), subtree stratum (5 m ≤*H* < 10 m), and shrub stratum (*H* < 5 m). Woody lianas were classified separately according to their climbing growth form, with species information obtained from iPlant (http://www.iplant.cn/). Stratum-specific NWP included biomass transfers associated with individuals moving between height-defined strata during the census interval.

Individuals were assigned to 10 DBH classes using 4-cm intervals starting at 1 cm, with an open-ended final class of *DBH* ≥37 cm, and six height classes using 5-m intervals, with a final class of *H* ≥ 25 m (**Supplementary Tables S1**, **S2**). DBH classes VIII–X represented *DBH* ≥ 29 cm, and height classes IV–VI represented *H* ≥15 m. Individuals were classified separately at each census using identical boundaries, so class-specific net changes included transfers across class boundaries.

### 2.3 Species diversity, stand structure, functional traits and phylogenetic diversity

All biodiversity, functional-trait, and stand-structure metrics were calculated at the 20 m × 20 m subplot scale using data from the 2016 census. These baseline variables characterized community conditions before the biomass changes observed during 2016–2021. Definitions, calculation procedures, and notation for all metrics are provided in **Supplementary Methods S1.3** and **Supplementary Table S3**.

#### 2.3.1 Species diversity and relative-abundance structure

Species diversity and relative-abundance structure were quantified using the Margalef richness index, Shannon–Wiener diversity index, Simpson diversity index, and Pielou evenness index. The Margalef index primarily describes species richness, whereas the Shannon–Wiener index incorporates both richness and evenness. The Simpson diversity index used here increases with diversity and gives greater weight to relatively common species; it was therefore used to characterize variation in species relative-abundance structure. Pielou evenness describes the distribution of individuals among species (Pielou, 1966; Simpson, 1949).

#### 2.3.2 Stand structural heterogeneity

Stand structural heterogeneity was characterized using indices of diameter-class diversity, species-by-size diversity, and local spatial structure (McElhinny et al., 2005; Pommerening, 2006). The *DBH*-based size diversity index (SDN) describes the distribution of individuals among diameter classes, whereas the species-by-size diversity index (SDNS) combines species identity and diameter-class membership. The stand spatial structural diversity index (DFS) integrates horizontal and vertical components, denoted by DFSh and DFSv, respectively. DFSh summarizes the diversity of neighborhood structural units, including their species composition, whereas DFSv characterizes vertical stratification (Buongiorno et al., 1994; Dong et al., 2024).

#### 2.3.3 Functional trait measurements and CWM traits

Functional traits were measured in 2016 to characterize the functional composition of the baseline community. Three healthy, mature individuals were selected for each sampled species. Three intact, fully expanded, sun-exposed mature leaves and four twig segments approximately 2–3 cm in diameter and 10 cm in length were collected from each individual. Samples were transported to the laboratory and processed following standardized protocols for plant functional trait measurements (Pérez-Harguindeguy et al., 2013). Detailed sampling and measurement procedures are provided in **Supplementary Methods S1.1**.

Six traits were measured: twig-derived wood density (WD, g·cm^-3^), twig dry matter content (TDMC, g·g^-1^), leaf area (LA, cm^2^), leaf thickness (LT, mm), specific leaf area (SLA, cm^2^·g^-1^), and leaf dry matter content (LDMC, g·g^-1^). WD was calculated as twig dry mass divided by fresh twig volume and was used as a proxy for stem wood density to reduce destructive sampling. TDMC was calculated as twig dry mass divided by twig fresh mass. LA was measured using a leaf area meter, and LT was measured using a digital caliper while avoiding the main vein. SLA was calculated as leaf area divided by leaf dry mass, and LDMC was calculated as leaf dry mass divided by leaf fresh mass. WD and TDMC represent tissue construction and resource-conservation properties, whereas LA, LT, SLA, and LDMC describe leaf morphology and resource-use strategies related to the leaf economics spectrum (Díaz et al., 2016; Wright et al., 2004).

Community-weighted mean (CWM) traits were calculated for each subplot from species-level mean trait values weighted by relative stem abundance. The resulting variables were CWM-WD, CWM-TDMC, CWM-LA, CWM-LT, CWM-SLA, and CWM-LDMC. These summarize trait composition across the included species, with greater weight given to species with higher stem abundance. Their interpretation was informed by the mass-ratio hypothesis (Díaz et al., 2007; Finegan et al., 2015; Grime, 1998).

Complete measurements of all six traits were available for 170 of the 195 woody species (87.2%), representing 43491 of the 43971 stems (98.9%) recorded in 2016. CWM traits and functional-diversity indices were calculated using the same set of 170 species. Within each subplot, relative abundance was calculated by dividing each included species’ stem count by the total stem count of the included species. These weights were applied consistently across all six CWM traits.

#### 2.3.4 Functional diversity

Functional diversity was calculated to describe interspecific differentiation in multidimensional trait space (Petchey and Gaston, 2006; Villéger et al., 2008), using the same 170 species with complete trait data described in Section 2.3.3. The six species-level mean traits were standardized to zero mean and unit variance across these species, and Euclidean distances were calculated in the standardized trait space.

Functional richness (FRic), functional evenness (FEve), functional divergence (FDiv), and Rao’s quadratic entropy (RaoQ) were calculated using the dbFD function in the FD package, with the 2016 subplot × species stem-abundance matrix restricted to the included species. FEve, FDiv, and RaoQ incorporated relative stem abundance, whereas FRic was independent of abundance. These metrics represent complementary aspects of trait-space occupancy, the abundance distribution of species within trait space, and pairwise functional dissimilarity among co-occurring species (Laliberté and Legendre, 2010; Rao, 1982; Villéger et al., 2008). Functional diversity was interpreted as a descriptor of trait dispersion and potential resource-use complementarity, rather than as direct evidence that complementarity occurred.

#### 2.3.5 Phylogenetic diversity

Phylogenetic metrics were used to describe species relatedness within each subplot (Cavender-Bares et al., 2009; Webb et al., 2002). Because a molecular phylogeny with empirical branch lengths was unavailable for all woody species in the plot, a rooted family–genus–species proxy tree was constructed from the available taxonomic classification. Branch lengths from the root to family, family to genus, and genus to species were each assigned a value of 1.

Five phylogenetic metrics were calculated for each subplot using the picante package: Faith’s phylogenetic diversity (Faith-PD), mean pairwise distance (MPD), mean nearest taxon distance (MNTD), net relatedness index (NRI), and nearest taxon index (NTI). Faith-PD represents the total branch length spanned by species within a community. MPD and MNTD describe the overall and nearest-neighbor phylogenetic distances among co-occurring species, respectively. NRI and NTI quantify phylogenetic clustering or overdispersion relative to null expectations (Faith, 1992; Webb et al., 2002). MPD and MNTD were calculated without abundance weighting, and NRI and NTI were calculated using the taxa.labels null model with 999 randomizations.

The proxy tree represented taxonomic hierarchy rather than estimated evolutionary divergence times. Consequently, these metrics were interpreted as taxonomy-based proxies for phylogenetic breadth and relatedness and could not capture variation in lineage ages, branch lengths, or unresolved evolutionary relationships.

### 2.4 Statistical analyses

Analyses used baseline community attributes from 2016 to explain net woody productivity during 2016–2021 across 150 subplots. Biomass differences between censuses were assessed using paired *t*-tests or Wilcoxon signed-rank tests, depending on the distribution of paired differences. Univariate regressions examined associations between individual predictors and productivity. Predictors were standardized before AIC-based stepwise multiple regression, with separate models fitted for the community, each height-defined stratum, and woody lianas. Model fit was summarized using R² and overall model tests. Standardized coefficients were interpreted as associations conditional on the other retained predictors.

Random forests ranked predictors retained in the multiple-regression models using 300 trees, the square-root rule for candidate predictors, and a minimum leaf size of three. Performance was assessed using training and out-of-bag R^2^, with additional shuffled five-fold cross-validation using 200-tree forests. Relative importance was calculated by normalizing the mean decrease in training R² across 50 permutations per predictor. Random seeds were fixed at 42. Permutation importance values were interpreted as exploratory rankings within the retained predictor set; they do not indicate the direction or statistical significance of individual predictor associations with productivity.

Principal component analysis was performed on standardized CWM-LT, CWM-SLA, and CWM-LA. PC1 explained 61.50% of their total variation and represented a leaf economics-related trait axis, with higher scores indicating higher CWM-SLA and CWM-LA but lower CWM-LT (**Supplementary Table S6**). Regression results, random-forest rankings, and ecological considerations informed the selection of Simpson diversity, DFSh, Faith-PD, RaoQ, and PC1 for the SEM. Before SEM fitting, pairwise Pearson correlations and variance inflation factors were assessed using |r| ≤0.70 and VIF < 3 as diagnostic criteria (**Supplementary Figure S2**; **Supplementary Table S7**).

A piecewise structural equation model incorporating the PCA-derived leaf-trait axis was used to assess direct and indirect associations with community NWP following the pathways specified in **Supplementary Table S4**. Global model fit was evaluated using the log-likelihood-based generalized chi-square test implemented in the LLchisq function of the piecewiseSEM package. Standardized path coefficients and component-model R^2^ values were reported. Indirect associations were calculated by summing coefficient products along all specified indirect pathways, including those containing nonsignificant coefficients. Total associations were the sum of direct and indirect components. Paths were interpreted as conditional associations rather than causal effects.

Global Moran’s *I* was calculated for community productivity and final community-regression residuals using row-standardized rook contiguity weights and 99,999 two-sided permutations (**Supplementary Table S9**). Regression, PCA, spatial diagnostics, and SEM analyses were performed in R using stats, MASS, car, spdep, and piecewiseSEM. Statistical significance was assessed at *p* < 0.05.

## 3 Results

### 3.1 Distribution of net woody productivity

Between 2016 and 2021, live woody biomass in the 6-ha Jinzi Mountain plot increased from 144.78 to 167.86 t·ha^-1^, corresponding to a mean net woody productivity of 4.62 t·ha^-1^·yr^-1^ (**Table 1**). The tree stratum accounted for most of the net increase, with biomass rising from 115.48 to 140.00 t·ha^-1^ and its share of total biomass increasing from 79.76% to 83.41%. Mean net woody productivity was 4.91 t·ha^-1^·yr^-1^ in the tree stratum, whereas the subtree and shrub strata showed negative values of −0.33 and −0.09 t·ha^-1^·yr^-1^, respectively. Woody lianas had a net woody productivity of 0.13 t·ha^-1^·yr^-1^. Biomass differed significantly between censuses at the community level and within each woody plant group (all *p* < 0.001).

**Table 1:** Biomass and net woody productivity by woody plant group.

Woody plant group	Biomass in 2016 (t·ha^-1^)	Biomass in 2021 (t·ha^-1^)	*p* value	Net woody productivity (t·ha^-1^·yr^-1^)
Tree stratum	115.48 ± 50.42	140.00 ± 54.93	< 0.001	4.91 ± 2.56
Subtree stratum	18.76 ± 11.80	17.10 ± 10.47	< 0.001	−0.33 ± 1.51
Shrub stratum	7.89 ± 4.06	7.44 ± 3.77	< 0.001	−0.09 ± 0.51
Woody liana	2.65 ± 3.09	3.31 ± 3.74	< 0.001	0.13 ± 0.16
Total	144.78 ± 46.04	167.86 ± 52.74	< 0.001	4.62 ± 2.37

Values are means ± standard deviations at the 20 m × 20 m subplot scale. Biomass and productivity are expressed as t·ha^-1^ and t·ha^-1^·yr^-1^, respectively. P values indicate differences in biomass between 2016 and 2021.

Net woody productivity varied markedly among *DBH* classes (**Figure 2a**). The biomass share of classes I–III declined from 20.70% to 19.18%, whereas that of classes VIII–X (*DBH* ≥ 29 cm) increased from 34.96% to 40.95%. Classes VIII–X contributed 78.53% of community net woody productivity. Class X (*DBH* ≥ 37 cm) alone contributed 2.03 t·ha^-1^·yr^-1^, or 44.02% of the total.

**Figure 2:**
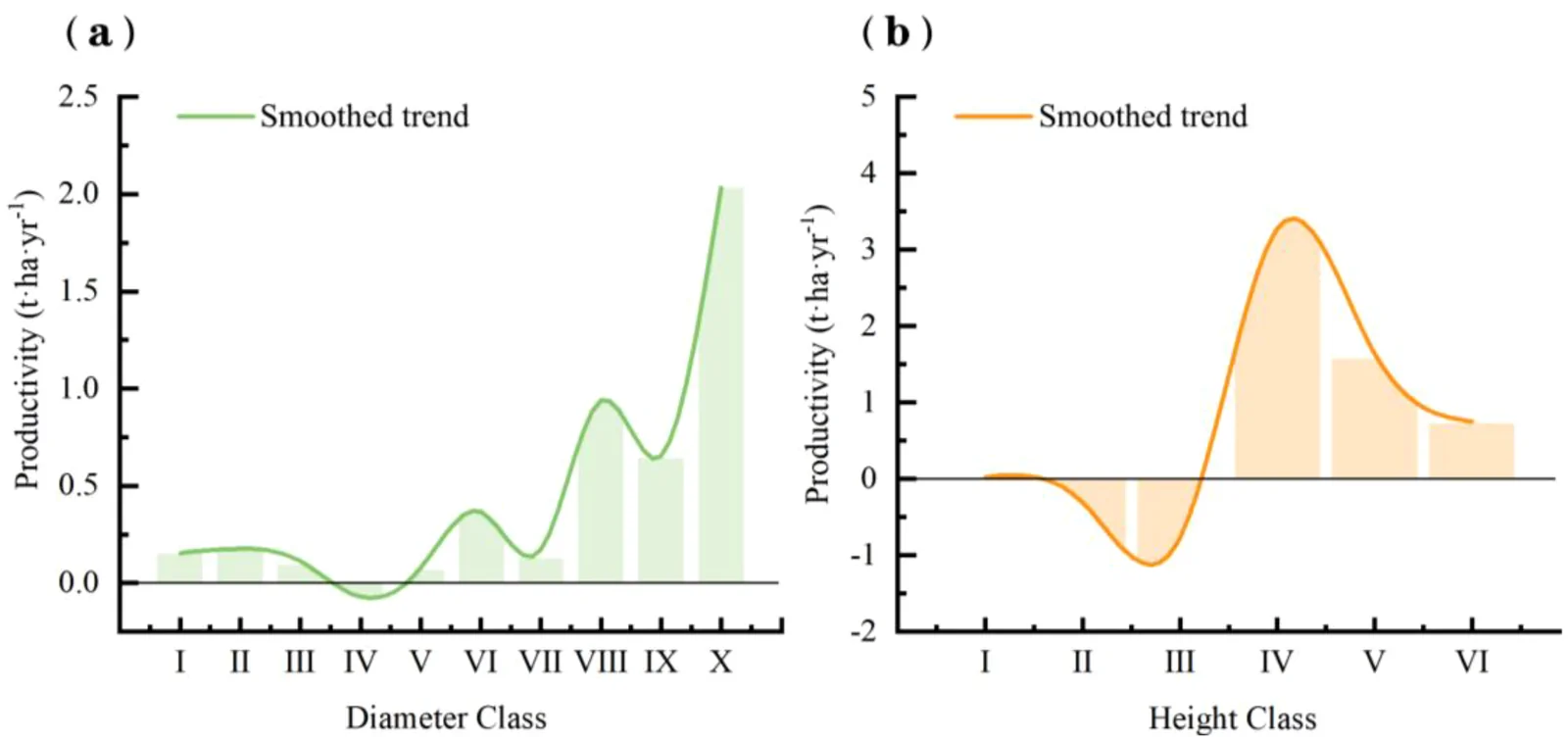
Net woody productivity across DBH and height classes. **(a)** DBH classes; **(b)** height classes. Bars show net woody productivity during 2016–2021; lines show smoothed trends. Height classes IV–VI together exceeded the community total because lower classes showed negative net biomass changes. Class boundaries are given in **Supplementary Tables S1**, **S2**.

The biomass share of height classes IV–VI (*H* ≥ 15 m) increased from 53.68% to 63.18% (**Figure 2b**). Class IV (15 m ≤ *H* < 20 m) contributed 3.28 t·ha^-1^·yr^-1^, accounting for 71.00% of community net woody productivity; classes V and VI contributed 1.64 and 0.75 t·ha^-1^·yr^-1^, respectively. Their combined contribution exceeded the community total because lower height classes showed negative net biomass changes.

### 3.2 Correlates of net woody productivity

Across subplots, community net woody productivity was positively associated with CWM-LT and CWM-LDMC and negatively associated with CWM-SLA and CWM-LA in univariate regressions (**Supplementary Figure S1**). Associations with CWM-LT and CWM-SLA were significant at *p* < 0.001, whereas those with CWM-LDMC and CWM-LA were significant at *p* < 0.05.

In the AIC-selected multiple-regression model, CWM-LT (β = 0.608), Simpson diversity (β = 0.661), SDNS (β = 0.651), and DFSh (β = 0.206) had significant positive coefficients, whereas RaoQ (β = −0.459), Pielou evenness (β = −0.743), and Margalef richness (β = −0.660) had significant negative coefficients (all *p* < 0.05). Faith-PD was retained but was not significant (β = 0.450, *p* ≥ 0.05). The model explained 34.0% of the variation in community net woody productivity (R^2^ = 0.340, *p* < 0.001; **Table 2**; **Supplementary Table S5**).

**Table 2:** AIC-selected regression models for net woody productivity.

Response variable	Retained predictors with positive β	Retained predictors with negative β	R^2^	Model *p*
Community productivity	CWM-LT (0.608***); Simpson (0.661**); SDNS (0.651**); DFSh (0.206*); Faith-PD (0.450, ns)	RaoQ (−0.459***); Pielou (−0.743**); Margalef (−0.660*)	0.340	<0.001
Tree-stratum NWP	CWM-LT (0.532***); DFS (0.297***); Simpson (0.890***)	Pielou (−0.585**); RaoQ (−0.312**)	0.226	<0.001
Subtree-stratum NWP	CWM-LDMC (0.343***); DFS (0.760**)	DFSv (−1.074***); CWM-WD (−0.182*)	0.215	<0.001
Shrub-stratum NWP	CWM-SLA (0.126, ns); MNTD (0.124, ns)	CWM-TDMC (−0.182, ns); DFSv (−0.169*)	0.070	<0.05
Woody liana NWP	Faith-PD (0.633**); CWM-WD (0.179*); Shannon–Wiener (0.854**)	CWM-LDMC (−0.490***); Margalef (−0.581*); CWM-TDMC (−0.196*); SDNS (−0.500*); CWM-SLA (−0.282*)	0.378	<0.001

Values are standardized coefficients (β) of AIC-retained predictors, conditional on the other predictors. *p < 0.05; **p < 0.01; ***p < 0.001; ns, *p* ≥ 0.05. R^2^ is the coefficient of determination; model p refers to the overall model test. Variable definitions are provided in **Supplementary Table S3**.

Within the retained predictor set, CWM-LT had the highest random-forest importance for community net woody productivity (22.75%), followed by Faith-PD (13.29%) and RaoQ (13.26%; **Figure 3**). CWM-LT also ranked first for the tree stratum (28.46%), followed by DFS and Simpson diversity. These rankings do not indicate association direction or statistical significance.

**Figure 3:**
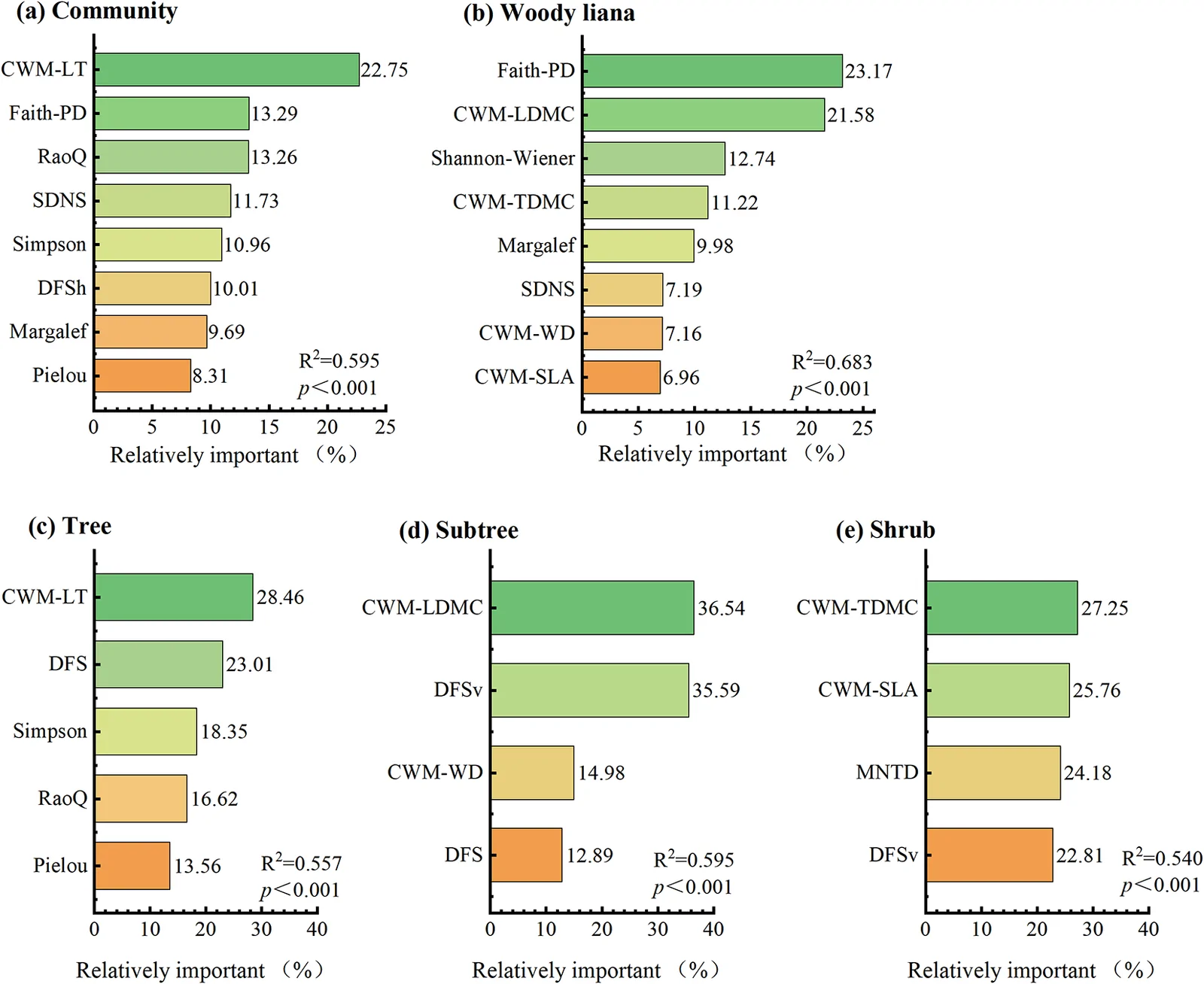
Random forest predictor importance for net woody productivity. Relative permutation importance of predictors retained in the corresponding multiple-regression models for **(a)** the community, **(b)** woody lianas, **(c)** the tree stratum, **(d)** the subtree stratum, and **(e)** the shrub stratum. *p* values refer to permutation tests of overall model performance, not the significance of individual predictors.

Moran’s *I* tests detected no significant global spatial autocorrelation in community net woody productivity (*I* = 0.077, *p* = 0.161) or final community-model residuals (*I* = 0.095, *p* = 0.092) under the specified spatial weights (**Supplementary Table S9**).

### 3.3 Direct and indirect associations with net woody productivity

The piecewise SEM showed no significant lack of fit (χ^2^ = 2.846, df = 4, *p* = 0.584), and its NWP component model explained 19.1% of the variation in community net woody productivity (R^2^ = 0.191; **Supplementary Table S8**). Species diversity (Simpson index) and structural diversity (DFSh) had significant positive direct associations with productivity (β = 0.278 and 0.198, respectively), whereas the leaf economics-related trait axis (PC1) had a significant negative direct association (β = −0.492; all *p* < 0.05). Direct associations with phylogenetic diversity (Faith-PD; β = 0.032) and functional diversity (RaoQ; β = −0.070) were not significant (both *p >* 0.05; **Figure 4a**).

**Figure 4:**
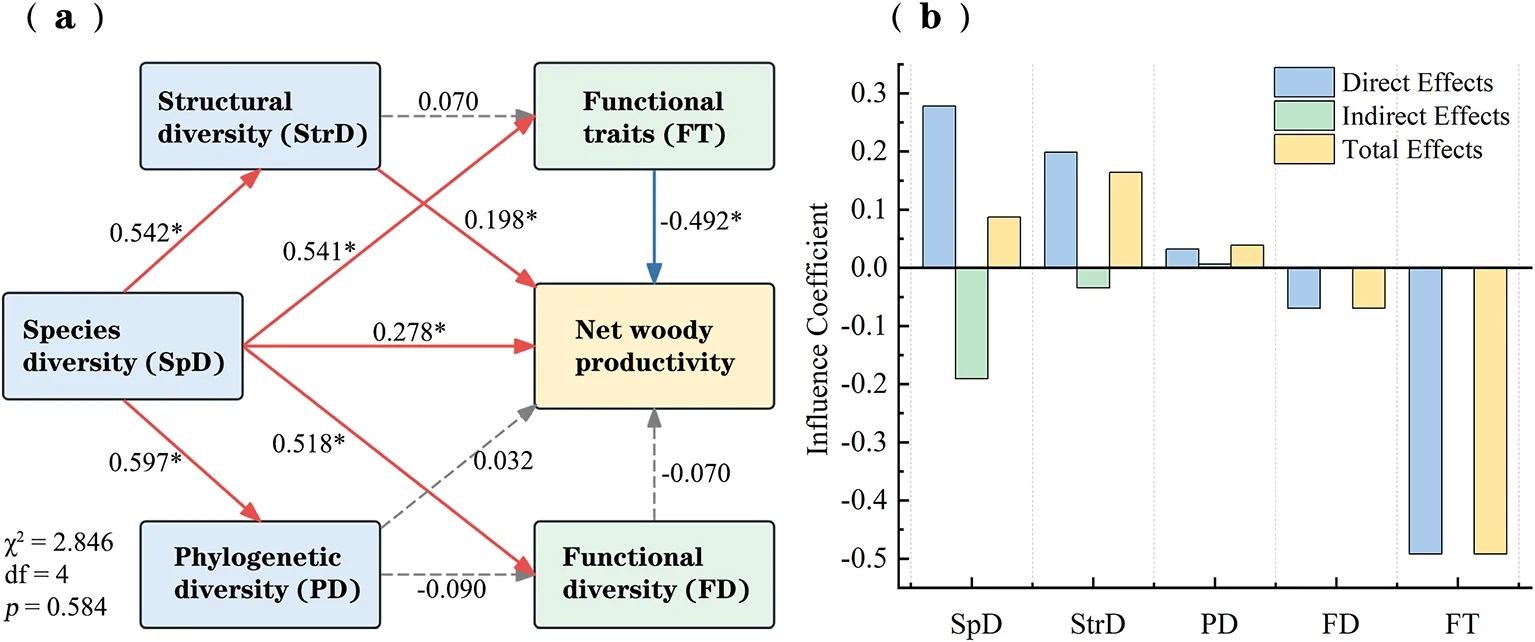
Direct and indirect pathways to community net woody productivity. **(a)** Piecewise SEM; **(b)** estimated direct, indirect, and total effects. Arrow labels are standardized coefficients. Solid red and blue arrows indicate significant positive and negative paths, respectively (*p < 0.05); dashed gray arrows indicate nonsignificant paths. SpD, Simpson diversity; StrD, DFSh; PD, Faith-PD; FD, RaoQ; FT, leaf-trait PC1, explaining 61.50% of variation in CWM-LT, CWM-SLA, and CWM-LA. Higher PC1 scores indicate higher CWM-SLA and CWM-LA but lower CWM-LT. Indirect effects sum coefficient products along all specified indirect pathways; total effects equal direct plus indirect effects.

PC1 explained 61.50% of the variation in CWM-LT, CWM-SLA, and CWM-LA, with higher scores indicating higher CWM-SLA and CWM-LA but lower CWM-LT (**Supplementary Table S6**). Species diversity was positively associated with structural diversity (DFSh), phylogenetic diversity, functional diversity, and PC1 (all *p* < 0.05; **Figure 4a**). Its positive direct association with productivity was partly offset by a negative net indirect association, primarily through PC1 (**Figure 4b**). The negative PC1–productivity path indicated that greater net woody productivity was associated with higher CWM-LT and lower CWM-SLA and CWM-LA within the specified model.

## 4 Discussion

### 4.1 Larger size classes accounted for most net woody productivity

Net woody productivity was concentrated in larger DBH and height classes, consistent with H1. Classes with *DBH* ≥ 29 cm contributed 78.53% of the community total, and those with *H* ≥15 m accounted for most net biomass accumulation. Gains in the tree stratum were partly offset by net biomass decreases in the subtree and shrub strata. Because individuals were reassigned to height strata and size classes at each census, these changes included transfers between groups. Lower-stratum decreases therefore cannot be attributed solely to mortality, and gains in larger classes do not represent the growth of a fixed cohort.

Absolute tree mass growth generally increased with size in a global analysis (Stephenson et al., 2014), whereas Lutz et al. (2018) documented the substantial share of forest biomass held by relatively few large trees. Large individuals also explained substantial variation in biomass stocks, gains, and losses in temperate forests of northeastern China (Yuan et al., 2021). In subtropical forests, large-tree attributes and size inequality were associated with biomass stocks (Wang et al., 2023), and tree-size diversity was associated with productivity (Ren et al., 2021). These findings support the relevance of tree size to forest carbon dynamics, although biomass stocks, individual growth, and stand-level net change represent different quantities.

Initial biomass and canopy position offer possible explanations for these size-related patterns. A larger initial biomass can yield a substantial absolute increment even at a lower relative growth rate, so greater biomass gains need not indicate higher physiological efficiency. Upper-canopy trees may also benefit from greater light interception and crown space (King et al., 2005), while size-dependent resource partitioning and asymmetric competition can influence stand growth (Forrester, 2019). These explanations have different management implications because retaining biomass stocks and modifying canopy structure may affect net woody productivity differently. Their relative contributions remain unresolved: growth was not assessed relative to initial biomass, and canopy light interception and competitive interactions were not measured.

Low mortality-related biomass losses in larger size classes could also have contributed to their positive net changes during the census interval. The balance between growth and mortality can strongly influence forest biomass dynamics (Bashir et al., 2019; Yuan et al., 2019). We did not compare size-specific mortality rates, so lower mortality among large trees remains an untested explanation. Conversely, the death of even a few large individuals could substantially reduce net biomass accumulation because each represents a large biomass loss.

The concentration of biomass stocks and net gains in larger individuals supports consideration of healthy large-tree retention and the recruitment of future canopy trees in management planning (Lutz et al., 2018; Ren et al., 2021). However, the observed distribution of net biomass change does not establish how the stand would respond to intervention. Whether canopy opening or selective removal would increase net woody productivity requires evaluation of survivor growth, recruitment, and mortality together.

### 4.2 Leaf-trait composition and structural diversity characterized productive subplots

The community regression was consistent with H2. CWM-LT, Simpson diversity, SDNS, and DFSh remained positively associated with net woody productivity after accounting for the other retained predictors, including Margalef richness (**Table 2**; **Supplementary Table S5**). CWM-LT also ranked first in the community and tree-stratum random forests fitted to the retained predictors. In the piecewise SEM, the leaf economics-related trait axis (PC1) showed a negative direct association with community net woody productivity.

The negative PC1–productivity relationship associated greater net woody productivity with thicker leaves, lower specific leaf area, and smaller leaf area. Lower SLA reflects greater dry-mass investment per unit leaf area and is consistent with the resource-conservative end of the leaf economics spectrum (Wright et al., 2004). Leaf thickness alone, however, is insufficient to identify a conservative strategy, and leaf size represents an additional dimension of plant form (Díaz et al., 2016). PC1 therefore captures variation related to leaf economics without representing the full spectrum. The relationship may also depend on stand conditions and resource availability (Forrester and Bauhus, 2016). Greater net biomass accumulation at lower PC1 scores could arise from the balance between growth and mortality and does not necessarily imply faster growth of conservative species.

The CWM–productivity relationships are compatible with the mass-ratio hypothesis, which links ecosystem functioning to the traits of dominant plants (Díaz et al., 2007; Finegan et al., 2015; Grime, 1998). Here, however, species were weighted by stem abundance. The resulting CWMs emphasize numerically abundant species, whose contributions to biomass and growth may differ from their contributions to stem counts. Our analysis therefore evaluates abundance-weighted functional composition but does not directly test the role of biomass-dominant species.

SDNS combines species identity and diameter-class heterogeneity, while DFSh characterizes horizontal variation in neighborhood structure. Their positive coefficients are consistent with differentiated occupation of growing space. Positive structural diversity–productivity relationships have also been reported in uneven-aged German forests (Dănescu et al., 2016) and across North American forests (LaRue et al., 2023). Zhai et al. (2024), however, found negative associations with biomass increment and net biomass change in US forests. In a subtropical tree-diversity experiment, Deng et al. (2025) linked canopy complexity to complementarity and biomass accumulation. Variation in structural metrics, stand development, and response definitions complicates comparisons among these studies. Our spatial indices did not measure three-dimensional canopy occupation or light interception, so the observed relationships cannot establish resource-use complementarity as the underlying mechanism.

The positive Simpson regression coefficient and direct piecewise SEM path associated greater diversity among relatively common species with higher net woody productivity. This pattern does not imply a benefit of stronger single-species dominance. In the SEM, negative indirect associations, mainly through leaf-trait PC1, partly offset the positive direct association, leaving a smaller positive total effect. Simpson diversity, Pielou evenness, and Margalef richness describe overlapping aspects of species abundance and entered the community regression together. Their contrasting coefficients are therefore conditional on the other retained predictors and provide no direct basis for predicting the consequences of manipulating richness or evenness (LaRue et al., 2023; Lutz et al., 2018).

### 4.3 Functional and phylogenetic diversity showed model-dependent associations with productivity

RaoQ was negatively associated with community net woody productivity in the multiple regression after accounting for CWM trait composition and stand structural heterogeneity; the Faith-PD coefficient was not significant. Neither metric had a significant direct path to productivity in the piecewise SEM, although both ranked relatively highly in the random forest. These rankings describe predictive importance within the selected variable set and do not indicate the direction or statistical significance of individual associations.

RaoQ summarizes abundance-weighted functional dissimilarity, but functional differentiation need not translate into complementary resource use (Laliberté and Legendre, 2010; Mammola et al., 2021). Complementarity depends on whether trait differences correspond to differences in resource acquisition or use under the prevailing conditions (Forrester and Bauhus, 2016; Loreau and Hector, 2001). Our leaf and twig traits may incompletely represent the canopy, hydraulic, and belowground differences relevant to woody biomass dynamics. Greater differentiation in the measured traits could therefore provide limited additional information after accounting for CWM composition and structural attributes. The negative regression coefficient is conditional on the other retained predictors and does not establish that functional differentiation reduced productivity. The nonsignificant direct path in the piecewise SEM further indicates that evidence for this relationship depends on model specification.

Liu et al. (2024) found that functional and phylogenetic diversity added little explanatory information beyond species richness in eastern US forests, with richness-independent metrics negatively related to productivity. By contrast, Hao et al. (2018) reported positive direct associations of functional dispersion and phylogenetic diversity with biomass increment at the 20 × 20 m scale in a temperate forest after accounting for initial biomass and topography. Their biomass-increment relationships weakened at larger spatial scales, and increment included survivor growth and recruitment rather than mortality-adjusted net change. They also used a broader trait set, including maximum height and leaf chemistry. These differences in response definition, trait representation, and covariate adjustment may help explain the contrast with our results, although their contributions cannot be isolated by comparing studies. The lack of a consistent biodiversity–productivity relationship across montane forest landscapes reported by Zou et al. (2024) further cautions against generalizing from one forest.

Faith-PD sums the branch lengths connecting species present in a community and, as applied here, does not weight species by abundance or biomass (Faith, 1992). Phylogenetic breadth may predict functioning when evolutionary distances capture relevant functional differences (Cadotte et al., 2008; Srivastava et al., 2012). This correspondence varies among traits and evolutionary depths (Paquette et al., 2015), and functional and phylogenetic diversity need not yield congruent community patterns (Cadotte et al., 2019). Hähn et al. (2025) documented widespread decoupling between these two dimensions in plant communities. Our taxonomy-derived proxy tree further limits interpretation because its equal branch lengths represent taxonomic hierarchy rather than measured evolutionary divergence. The nonsignificant Faith-PD coefficient and direct SEM path therefore do not demonstrate that evolutionary history is unimportant for forest productivity.

Nonsignificant direct paths also do not exclude other ecological roles. Species diversity was positively associated with functional and phylogenetic diversity in the SEM, but these relationships did not establish causal mediation of productivity. Longer-term evidence from Canadian forests links greater functional, phylogenetic, and taxonomic diversity to greater temporal stability of productivity (Ding et al., 2024). Phylogenetic diversity also represents conservation information beyond short-term biomass accumulation (Cardillo, 2023). Our results therefore do not justify prioritizing productive trait combinations at the expense of functional or phylogenetic diversity. Retaining diverse assemblages together with healthy canopy trees and structural heterogeneity may accommodate several management objectives, although the productivity consequences require further testing.

### 4.4 Study limitations and future directions

The omission of initial biomass, basal area, and large-tree attributes is a major limitation. Subplots with greater initial biomass may show larger absolute biomass gains even when relative growth rates are similar. Covariation between initial biomass and the included predictors could therefore contribute to their observed associations (Bashir et al., 2019). Initial biomass has been identified as an important predictor of biomass growth in temperate forests (Yuan et al., 2019). Our analyses cannot separate this explanation from trait or structural associations. Explicit adjustment for baseline stocks and size structure, together with separate models of growth, recruitment, and mortality, would clarify their contributions to net woody productivity.

Inference is also restricted by the single-plot design and five-year interval. The 150 contiguous subplots characterize variation within this forest, and nonsignificant global Moran’s *I* tests do not exclude local spatial dependence. Species-mean traits, stem-abundance weighting, incomplete trait coverage, and the taxonomy-derived phylogeny further limit functional and evolutionary interpretation.

The SEM describes associations under the specified pathway structure. Model fit alone cannot establish causal direction or exclude unmeasured environmental and historical confounding (Byrnes and Dee, 2025). Validation in independent plots is needed because predictor selection and subsequent path analysis used the same dataset. Longer records with more frequent censuses across stand developmental stages and environmental gradients would help assess whether these associations persist through time and across sites. Combining demographic analyses with measurements of baseline biomass, canopy structure, and environmental conditions would help distinguish their possible explanations.

## 5 Conclusions

During 2016–2021, net woody productivity in the Jinzi Mountain subtropical mixed forest was concentrated in the tree stratum and larger diameter and height classes. Across subplots, net woody productivity was associated with stem-abundance-weighted leaf-trait composition, species relative-abundance structure, and stand structural heterogeneity. Univariate relationships indicated greater productivity in subplots with higher CWM-LT and lower CWM-SLA and CWM-LA, consistent with the direction of the leaf-trait PC1 path in the piecewise SEM. Species diversity and structural diversity had significant positive direct paths to productivity in the piecewise SEM. Evidence for functional diversity depended on model specification: RaoQ was negatively associated with productivity in the multiple regression but had a nonsignificant direct SEM path. Phylogenetic diversity also had a nonsignificant direct path.

Because initial biomass was not included in the models, these associations may partly reflect differences in baseline biomass stocks. Retaining healthy large canopy trees, woody-species diversity, and heterogeneous stand structure merits consideration in close-to-nature forest management, although the effects of these practices on net woody productivity remain to be tested.

## Data Availability

The raw data supporting the conclusions of this article will be made available by the authors, without undue reservation.
