# Supplementary material for: Leaf economic strategy and stand structural complexity are associated with net woody productivity in a subtropical mixed forest
Source: Front Plant Sci. 2026 Oct 6;17:1980200. doi: 10.3389/fpls.2026.1980200 (PMC13680441; doi:10.3389/fpls.2026.1980200)
Supplement: Supplementary file 1 [file SupplementaryFile1.docx]

## Supporting Information

### S1 Methodological Supplement

#### S1.1 Functional trait sampling and measurement

Functional traits were measured in 2016 to characterize the initial functional structure of the 6-ha Jinzi Mountain plot. Based on species composition and dominance patterns within the plot, mature, healthy, and representative woody individuals were selected for trait sampling. For each sampled species, three individuals were selected, and three fully expanded, undamaged, sun-exposed mature leaves were collected from each individual. Four branch segments approximately 2–3 cm in diameter and 10 cm in length were also collected from each individual. Leaf and branch samples were transported to the laboratory for trait measurements following standardized protocols for plant functional traits (Cornelissen et al., 2003; Pérez-Harguindeguy et al., 2013).

Six functional traits were used in this study, including two stem-related traits and four leaf traits. Wood density (WD, g·cm^-3^) was calculated as branch dry mass divided by branch volume. To reduce destructive sampling in the permanent plot, branch density was used as a proxy for WD. Twig dry matter content (TDMC, g·g^-1^) was calculated as twig dry mass divided by twig fresh mass. Leaf area (LA, cm^2^) was measured using a leaf area meter, and leaf thickness (LT, mm) was measured with a digital caliper while avoiding the main vein. Specific leaf area (SLA, cm^2^·g^-1^) was calculated as leaf area divided by leaf dry mass, and leaf dry matter content (LDMC, g·g^-1^) was calculated as leaf dry mass divided by leaf fresh mass (Cornelissen et al., 2003; Pérez-Harguindeguy et al., 2013).

WD and TDMC describe aspects of tissue construction and dry matter investment, whereas LA, LT, SLA, and LDMC characterize leaf size, light capture, tissue investment, and variation along the leaf economics spectrum (Chave et al., 2009; Díaz et al., 2016; Wright et al., 2004). Community-weighted mean (CWM) traits, including CWM-WD, CWM-TDMC, CWM-LA, CWM-LT, CWM-SLA, and CWM-LDMC, were calculated using the same set of 170 woody species with complete measurements of all six traits. Within each 20 × 20 m subplot, each species-level mean trait value was weighted by that species’ 2016 stem count divided by the total stem count of the included species present in the subplot. The same species weights were applied to all six traits within each subplot, and trait values were retained in their original measurement units. These CWM traits represented stem-abundance-weighted trait composition, and their interpretation was informed by the mass-ratio hypothesis (Díaz et al., 2007; Finegan et al., 2015; Grime, 1998).

#### S1.2 Biomass estimation and classification of height strata, woody lianas, and size classes

Individual live woody biomass was estimated for the 2016 and 2021 censuses using the allometric equation dataset compiled for subtropical mixed evergreen–deciduous broad-leaved forests (Wu et al., 2022). For each woody individual with DBH ≥1 cm, species identity was used to select an applicable equation. Species-specific equations were preferentially applied; when these were unavailable, general equations for the corresponding vegetation layer were used following Wu et al. (2025). Measured *DBH*, height (*H*), and crown width (*CW*) were entered according to the predictor variables required by the selected equation, with measurement units matched to those specified in the equation. Biomass was estimated separately from the measurements recorded in each census.

Individual biomass estimates were summed within each 20 × 20 m subplot and converted to t·ha^-1^. With individual biomass expressed in kilograms, this conversion was calculated as:

$$B_{t}\text{=}\frac{\sum_{i} b_{i,t}}{1000A}=0.025\sum_{i} b_{i,t}$$

where *B_t_* is subplot live woody biomass per unit area in census year t (t·ha^-1^), *b_i,t_* is the biomass of live individual i in that census (kg), and *A* is the subplot area (0.04 ha). The summation includes all live woody individuals meeting the census threshold, and 1000 converts kilograms to tonnes.

Net woody productivity (NWP) was calculated as:

$$\text{NWP}\text{=}\frac{B_{2021}-B_{2016}}{5}$$

where *B_2016_* and *B_2021_* are live woody biomass per unit area in the respective censuses, and 5 is the census interval in years. NWP was expressed as t·ha^-1^·yr^-1^. Individual identification numbers were used to match records between censuses. Surviving individuals contributed their biomass estimates to both census totals; recruits contributed only to the 2021 total; and individuals that died during the interval contributed only to the 2016 total. The difference between census biomass totals therefore incorporated survivor growth, recruitment, and mortality without separate adjustments for recruitment gains or mortality losses.

NWP represented the annualized net change in standing live woody biomass; litterfall, fine-root production, and reproductive allocation were not included (Clark et al., 2001; Malhi et al., 2004).

At each census, self-supporting woody individuals were assigned to three height-defined strata: the tree stratum (H ≥10 m), subtree stratum (5 m ≤H <10 m), and shrub stratum (H <5 m). Woody lianas were classified separately according to their climbing growth form, with species information obtained from iPlant (http://www.iplant.cn/). Woody species were also classified as evergreen or deciduous according to leaf phenology.

Individuals were grouped into 10 *DBH* classes using 4-cm intervals for the bounded classes. The first class was 1 cm ≤ *DBH* <5 cm, and the final, open-ended class was *DBH* ≥37 cm (Table S1). Previous studies have shown that 4-cm diameter-class intervals can effectively characterize the relationship between tree size differentiation and forest growth (Lei et al., 2009). Individuals were grouped into six height classes using 5-m intervals for the bounded classes. The first class was *H* <5 m, and the final, open-ended class was *H* ≥25 m (Table S2). The same class boundaries were applied in both censuses.

Biomass and NWP were summarized for the height-defined strata and woody lianas, and separately by DBH and height class. Group-specific NWP was calculated from the difference between the biomass totals assigned to each group in the two censuses. Values for height strata and size classes included biomass transfers across boundaries and did not represent the growth of a fixed cohort. Negative net changes in some groups allowed the combined contribution of other groups to exceed the community total.

Table S1 DBH class boundaries (cm)

| DBH class | Ⅰ | Ⅱ | Ⅲ | Ⅳ | V | Ⅵ | Ⅶ | Ⅷ | Ⅸ | Ⅹ |
| --- | --- | --- | --- | --- | --- | --- | --- | --- | --- | --- |
| DBH | [1,5) | [5,9) | [9,13) | [13,17) | [17,21) | [21,25) | [25,29) | [29,33) | [33,37) | [37,+∞) |

Table S2 Height class boundaries (m)

| Height class | Ⅰ | II | III | IV | V | VI |
| --- | --- | --- | --- | --- | --- | --- |
| *H* | [0,5) | [5,10) | [10,15) | [15,20) | [20,25) | [25,+∞) |

#### S1.3 Calculation of biodiversity metrics

Net woody productivity and community attributes were calculated for each 20 × 20 m subplot. All explanatory metrics were derived from the 2016 census to characterize community conditions preceding biomass change during 2016–2021. The formulas, symbols, ecological meanings, and references for biodiversity, stand structure, and community-weighted mean trait metrics are provided in Table S3.

Species-diversity indices were calculated from the 2016 subplot × species stem-abundance matrix. Species richness and diversity indices were obtained using the vegan package, whereas the Margalef and Pielou indices were calculated from species richness, total stem abundance, and the Shannon index using base R. Structural-diversity indices were calculated from 2016 DBH, height, species identity, and stem-coordinate data. SDN and SDNS were derived from the predefined DBH-class distribution, whereas DFS, DFSh, and DFSv were calculated using custom R functions based on the structural-unit algorithm described by Hui et al. (2023) and Dong et al. (2024).

Functional diversity was calculated for 170 woody species with complete measurements of all six traits described in Section S1.1. These species accounted for 43,491 of the 43,971 censused woody individuals (98.91%). Species-level mean trait values were standardized to zero mean and unit variance before analysis, and Euclidean distances were calculated among species in the standardized trait space. Functional richness (FRic), functional evenness (FEve), functional divergence (FDiv), and Rao’s quadratic entropy (RaoQ) were calculated using the dbFD function in the FD package. The 2016 subplot × species abundance matrix, restricted to these 170 species, was supplied as the abundance input. FEve, FDiv, and RaoQ incorporated relative stem abundance, whereas FRic was independent of abundance. RaoQ was not standardized by its theoretical maximum. CWM traits were calculated using the same species set. Within each subplot, species weights were calculated relative to the total stem abundance of the included species, and trait values were retained in their original measurement units.

Phylogenetic diversity was estimated from a taxonomy-derived proxy phylogeny because a molecular phylogeny with empirical branch lengths was unavailable for the woody species in the plot. A rooted family–genus–species tree was constructed from the taxonomic classification table. Branch lengths from the root to family, family to genus, and genus to species were each set to 1. Thus, the distance between congeneric species was 2, between species from different genera within the same family was 4, and between species from different families was 6. These distances represented taxonomic separation on the proxy tree rather than evolutionary time. Faith’s PD, MPD, MNTD, NRI, and NTI were calculated using the picante package. Faith’s PD was calculated from the subplot species-incidence matrix and the proxy tree, whereas MPD and MNTD were calculated from the cophenetic distance matrix without abundance weighting. NRI and NTI were calculated as the negative standardized effect sizes of MPD and MNTD, respectively, using the taxa.labels null model with 999 randomizations.

Table S3 Biodiversity, stand-structure, and community-weighted trait metrics used in this study

| **Category** | **Metric** | **Abbreviation** | **Formula** | **Formula notation** | **Ecological meaning** | **Main references** |
| --- | --- | --- | --- | --- | --- | --- |
| Species diversity | Margalef richness index | Margalef | $\text{D}_{\text{Mg}}\text{=}\frac{\text{S}\text{-}\text{1}}{\ln\text{N}}$ | *S* is the number of species within a subplot, and *N* is the total number of woody individuals within that subplot. | Represents species richness relative to the logarithm of stem abundance | (Ulanowicz, 2001) |
|  | Shannon-Wiener diversity index | Shannon | $\text{H}^{\text{'}}\text{=}\text{-}\sum_{i=1}^{S} \text{P}_{\text{i}}\text{ln} \text{P}_{\text{i}}$ | *S* is the number of species within a subplot; *p_i_* is the proportion of individuals belonging to species i relative to the total number of individuals within the subplot. | Reflects both species richness and evenness | (Shannon, 1948; Ulanowicz, 2001) |
|  | Simpson diversity index | Simpson | $\text{D}\text{=1}\text{-}\sum_{i=1}^{S} {\text{P}_{\text{i}}}^{\text{2}}$ | *p_i_* is the relative abundance of species i; *S* is the number of species within a subplot. | Sensitive to dominant species and relative abundance structure | (Simpson, 1949) |
|  | Pielou evenness index | Pielou | $\text{J}\text{=}\frac{\text{H}^{\text{'}}}{\ln\text{S}}$ | *S* is the number of species within a subplot. | Represents the evenness of individuals among species | (Pielou, 1966) |
| Structural diversity | Size diversity based on DBH | SDN | $\text{SDN}\text{=}\text{-}\sum_{\text{j}\text{=1}}^{\text{d}_{\text{c}}} \frac{\text{n}_{\text{j}}}{N}\text{ln(}\frac{\text{n}_{\text{j}}}{N}\text{)}$ | *d_c_* is the number of diameter classes within a subplot; *n_j_* is the number of individuals in the j-th diameter class; *N* is the total number of individuals within the subplot. Terms with zero abundance contribute zero. | Describes heterogeneity in diameter distribution and stem size differentiation | (Buongiorno et al., 1994) |
|  | Size diversity based on DBH and species identity | SDNS | $\text{SDNS}\text{=}\text{-}\sum_{\text{i}\text{=1}}^{\text{n}_{\text{sp}}} \sum_{\text{j}\text{=1}}^{\text{d}_{\text{c}}} \frac{\text{n}_{\text{ij}}}{N}\text{ln(}\frac{\text{n}_{\text{i}\text{j}}}{N}\text{)}$ | *d_c_* is the number of diameter classes; *n_sp_* is the number of species within the subplot; *n_i_*_j_ is the number of individuals of species i in diameter class j; *N* is the total number of individuals within the subplot. Terms with zero abundance contribute zero. | Describes combined heterogeneity in species identity and diameter structure | (Buongiorno et al., 1994) |
|  | Stand spatial structural diversity index | DFS | $\text{D}_{\text{FS}}\text{=}\frac{\text{1}}{\text{2}}\text{(}\text{D}_{\text{FS}\text{h}}\text{+}\text{D}_{\text{FSv}}\text{)}$ | *DFS_h_* and *DFS_v_* denote horizontal and vertical structural diversity, respectively; structural units are defined based on neighborhood relationships, species composition, and forest strata information. | Represents overall spatial structural complexity of the stand | (Dong et al., 2024; Hui et al., 2023) |
|  | Horizontal structural diversity | DFSh | $\text{D}_{\text{FS}\text{h}}\text{=}\frac{\text{1}}{\text{2}}\text{\{}\frac{\text{1}}{\text{2}}\text{[(1-}\frac{\text{1}}{\text{2}}\sum_{\text{i}\text{=1}}^{\text{N}_{\text{s}}} \text{\vert}\text{x}_{\text{i}}\text{-}\text{y}_{\text{i}}\text{\vert}\text{)}$  $\text{+}\frac{\text{ln}\text{N}_{\text{s}}}{\text{ln}\text{625}}\text{]+}\frac{\text{ln}\text{S}_{\text{n}}}{\text{ln}\text{5}}\text{\}}$ | *N_s_* is the number of horizontal structural unit types; *x_i_* is the relative frequency of the i-th type; *y_i_* is the theoretical frequency under a uniform distribution; *S_n_* is the mean number of tree species per structural unit. | Represents horizontal spatial configuration and neighborhood heterogeneity | (Dong et al., 2024; Hui et al., 2023) |
|  | Vertical structural diversity | DFSv | $\text{D}_{\text{FSv}}\text{=}\frac{\text{ln}\text{N}_{\text{cm}}}{\text{ln}\text{3}}$ | *N_cm_* is the mean number of forest strata represented within a structural unit. | Represents vertical stratification and height structural complexity | (Dong et al., 2024; Hui et al., 2023) |
| Phylogenetic diversity | Faith’s phylogenetic diversity | Faith-PD | $\text{PD}\text{=}\sum_{b\in\beta}^{\text{q}} L_{b}$ | *β* is the set of branches represented by the species present in the subplot, according to the root-inclusion setting used in the calculation. *L_b_* is the length of branch *b* on the taxonomy-derived proxy phylogeny. | Summarizes the branch length represented by subplot species on the taxonomy-derived proxy phylogeny | (Faith, 1992) |
|  | Mean pairwise phylogenetic distance | MPD | $\text{MPD}\text{=}\frac{2}{S(S-1)}\sum_{i=1}^{S-1} \sum_{\text{j=}\text{i}\text{+}\text{1}}^{S} d_{\text{i}\text{j}}$ | *S* is the number of woody species within the subplot, and *d_ij_* is the phylogenetic distance between species *i* and *j* on the taxonomy-derived proxy phylogeny. | Represents the mean phylogenetic distance among all species pairs | (Webb et al., 2002) |
|  | Mean nearest taxon distance | MNTD | $\text{MNTD}\text{=}\frac{\sum_{\text{i}\text{=1}}^{\text{N}_{\text{sp}}} \text{p}_{\text{imin}}}{\text{N}_{\text{sp}}}$ | *p_imin_* denotes the nearest phylogenetic distance between tree species i and all species within the subplot; *N_sp_* is the number of tree species within the subplot. | Represents the mean nearest-neighbor phylogenetic distance | (Webb et al., 2002) |
|  | Net relatedness index | NRI | $\text{NRI}\text{=}\text{-}\frac{\text{MPD}\text{-}\text{MPD}_{\text{r}}}{{\text{SD}\text{(}\text{MPD}}_{\text{r}}\text{)}}$ | *MPD_r_* is the mean *MPD* obtained from randomization; *SD(MPD_r_)* is the standard deviation of randomized *MPD*. | Indicates phylogenetic clustering or overdispersion based on whole-community phylogenetic distance | (Webb et al., 2002) |
|  | Nearest taxon index | NTI | $\text{NTI}\text{=}\text{-}\frac{{\text{MNTD}\text{-}\text{MNTD}}_{\text{r}}}{\text{SD}\text{(}\text{MNTD}_{\text{r}}\text{)}}$ | *MNTD_r_* is the mean *MNTD* obtained from randomization; *SD(MNTD_r_)* is the standard deviation of randomized *MNTD*. | Indicates phylogenetic clustering or overdispersion near the tips of the phylogeny | (Webb et al., 2002) |
| Functional diversity | Functional richness | FRic | $\text{F}\text{R}_{\text{ic}}\text{=}\frac{V_{\mathrm{subplot}}}{V_{pool}}$ | *V_subplot_* is the convex hull volume occupied by species within the subplot; *V_pool_* is the convex hull volume occupied by all included species in the same functional space. | Represents the convex hull volume occupied by species in multidimensional functional space | (Villéger et al., 2008) |
|  | Functional evenness | FEve | $\text{F}\text{E}_{\text{ve}}\text{=}\frac{\sum_{\text{e}\text{=1}}^{\text{S}} \text{min}\text{(}\text{P}_{e}\text{,}\frac{\text{1}}{\text{S}}\text{)}\text{-}\frac{1}{S-1}}{1-\frac{1}{S-1}}$  $P_{e}\text{=}\frac{\frac{d_{ij}^{F}}{P_{i}+P_{j}}}{\sum_{\text{r}\text{=1}}^{\text{S}\text{-1}} {EW}_{r}}$ | *S* is the number of included species present in the subplot. Edge *e* connects species *i and j* in the minimum spanning tree constructed from functional distances. *d^F^_ij_* is their functional distance, and *p_i_* and *p_j_* are their relative stem abundances. *P_e_* is its normalized value | Represents the evenness of species distribution in functional trait space, accounting for interspecific distances and relative abundances | (Villéger et al., 2008) |
|  | Functional divergence | FDiv | $\text{F}\text{D}_{\text{iv}}\text{=}\frac{\sum_{i=1}^{S} q_{i}(p_{i}-\bar{p})+\bar{p}}{\sum_{i=1}^{S} q_{i}\vert p_{i}-\bar{p}\vert+\bar{p}}$  $p_{i}=\vert\vert u_{i}-g\vert\vert$  $\bar{p}\text{=}\frac{1}{S}\sum_{i=1}^{S} p_{i}$ | *u_i_* is the coordinate vector of species *i* in the retained functional space; *g* is the arithmetic mean of the convex hull vertex coordinates. *p_i_* is the Euclidean distance from species *i* to *g*; $\bar{p}$ is the unweighted mean of these distances across the *S* included species present in the subplot; and $q_{i}$ is the relative stem abundance of species i. | Represents the extent to which abundant species occur farther from the centroid of the convex hull vertices | (Villéger et al., 2008) |
|  | Rao’s quadratic entropy | RaoQ | $\text{F}\text{D}_{\text{iv}}\text{=}\sum_{i=1}^{S-1} \sum_{j=i+1}^{S} p_{i}p_{j}{(d_{ij}^{F})}^{2}$  $d_{ij}^{F}=\sqrt{\sum_{k=1}^{n_{r}} {(z_{ik}-z_{jk})}^{2}}$  $z_{ik}=\frac{x_{ik}-\bar{x}_{k}}{S_{k}}$ | *S* is the number of included species present in the subplot; *p_i_* and *p_j_* are their relative stem abundances*. d^F^_ij_* is the Euclidean distance between species *i* and *j* based on standardized traits. *x_ik_* is the mean value of trait *k* for species *i;* $\bar{x}_{k}$ *and S_k_* are the mean and standard deviation of that trait across all 170 included species; and *n_T_=6* | Integrates species relative abundance and pairwise functional dissimilarity | (Laliberté and Legendre, 2010; Rao, 1982) |
| Functional trait composition | Community-weighted mean trait | CWM | $\text{CWM}\text{.}\text{x}\text{=}\sum_{\text{i}\text{=1}}^{\text{S}} \text{p}_{\text{i}}\text{∙}\text{x}_{\text{i}}$ | *p_i_* is the relative abundance of tree species i within a subplot; *x_i_* is the value of a given functional trait for tree species i within the subplot. | Describes community trait composition weighted by relative stem abundance | (Díaz et al., 2007; Grime, 1998) |

**Note:** All explanatory metrics were calculated using the 2016 community data. Functional diversity and CWM traits were calculated for the same 170 woody species with complete measurements of all six traits. Relative-abundance weights were based on the total stem count of these species within each subplot. Phylogenetic metrics were derived from a taxonomy-based proxy tree with assigned branch lengths and therefore represent taxonomic relationships rather than empirical evolutionary times. CWM values retained the original trait units, and the same species weights were used for all six CWM traits within each subplot. Abbreviations and measurement units for individual traits are provided in Section S1.1.

#### S1.4 Variable screening, PCA, and model fitting

All explanatory variables represented community conditions in 2016. Univariate linear regressions first examined associations between individual predictors and net woody productivity at the community level and separately for the tree, subtree, and shrub strata and woody lianas. Candidate predictors were standardized before stepwise selection of multiple linear regression models based on Akaike’s information criterion (AIC). Separate models were fitted for the community and each woody plant group. Model performance was summarized using ordinary R^2^ and overall model tests, and standardized coefficients were interpreted as associations conditional on the other predictors retained in the same model. Pairwise correlations and variance inflation factors were not used as an initial screening step for these regression models.

Random forests were subsequently used to rank predictors retained in the corresponding multiple-regression models. Each random forest comprised 300 trees. The number of candidate predictors at each split followed the square-root rule, and each terminal node contained at least three observations. Model performance was summarized using training and out-of-bag R^2^. Additional five-fold cross-validation used randomly shuffled folds and 200-tree forests with otherwise identical settings. Permutation importance was calculated from the mean decrease in training-set R² across 50 permutations of each predictor and expressed as a percentage of the summed mean decreases across all predictors in the corresponding model. Random seeds were fixed at 42. Random forests were used for exploratory importance ranking, whereas association direction was determined from the regression models.

Principal component analysis (PCA) was performed on standardized CWM-LT, CWM-SLA, and CWM-LA to construct a parsimonious leaf-trait axis for the structural equation model. The first principal component (PC1) explained 61.50% of their total variation and was retained as a leaf economics-related trait axis. Higher PC1 scores indicated higher CWM-SLA and CWM-LA but lower CWM-LT (Table S6).

Before SEM fitting, pairwise Pearson correlations and variance inflation factors were examined among Simpson diversity, DFSh, Faith-PD, RaoQ, and PC1. Absolute correlations ≤0.70 and VIF values <3 were considered acceptable (Figure S2; Table S7). A piecewise structural equation model incorporating the PCA-derived trait axis was then used to assess direct and indirect associations between community attributes and net woody productivity. Species diversity, structural diversity, phylogenetic diversity, functional diversity, and functional trait composition were represented by Simpson diversity, DFSh, Faith-PD, RaoQ, and PC1, respectively.

The component models specified DFSh and Faith-PD as functions of Simpson diversity; PC1 as a function of Simpson diversity and DFSh; RaoQ as a function of Simpson diversity and Faith-PD; and community net woody productivity as a function of Simpson diversity, DFSh, Faith-PD, RaoQ, and PC1. Component relationships were fitted using ordinary least squares. Global model fit was evaluated using the log-likelihood-based generalized chi-square test implemented in LLchisq. This test compares the summed log-likelihoods of the specified component models with those of the corresponding saturated models. Explanatory power was summarized using component-model R^2^ values. Standardized path coefficients were reported. Indirect associations were calculated by multiplying coefficients along each directed pathway and summing these products when multiple indirect pathways linked the same variables. Total associations were calculated as the sum of direct and indirect components. These coefficients were interpreted as conditional associations and did not establish causal effects. The ecological rationale for the hypothesized pathways is summarized in Table S4.

Global Moran’s I was calculated for community net woody productivity and residuals from the final community multiple-regression model using row-standardized rook-contiguity weights. Subplots were defined as neighbors when they shared a 20-m edge, and statistical significance was evaluated using 99999 unrestricted two-sided permutations (Table S9).

Linear regressions, PCA, piecewise SEM, and spatial-autocorrelation analyses were performed in R 4.4.0 using the stats, MASS, car, piecewiseSEM, and spdep packages. Random-forest analyses were performed in Python using scikit-learn.

Table S4 Ecological rationale for SEM pathways

| **Hypothesized pathway** | **Pathway meaning** | **Ecological mechanism or hypothesis** | **Main references** |
| --- | --- | --- | --- |
| Species diversity  →  Structural diversity | Covariation between species diversity and horizontal structural heterogeneity. | Species differences in growth form, shade tolerance, and canopy position may contribute to variation in neighborhood structure. | (Ehbrecht et al., 2021; Perles-Garcia et al., 2021) |
| Species diversity  →  Phylogenetic diversity | Covariation between species diversity and taxonomic breadth. | Species richness and composition contribute to both Simpson diversity and the branch length represented on the taxonomy-derived proxy tree. | (Faith, 1992; Webb et al., 2002) |
| Species diversity  →  Functional diversity | Covariation between species diversity and abundance-weighted functional dissimilarity. | Changes in species composition and relative abundance alter the representation of trait differences within a community. | (Petchey and Gaston, 2006; Villéger et al., 2008) |
| Species diversity  →  Functional traits | Association between species diversity and community-weighted leaf trait composition. | Changes in the relative abundances of species with contrasting leaf traits can shift community-weighted trait values. | (Díaz et al., 2007; Grime, 1998) |
| Species diversity  →  Net woody productivity | Association between species diversity and net woody productivity conditional on the other modeled attributes. | Selection effects and resource complementarity provide possible explanations for diversity–productivity relationships. | (Liang et al., 2016; Loreau and Hector, 2001) |
| Structural diversity  →  Functional traits | Association between horizontal structural heterogeneity and community-weighted leaf trait composition. | Differences in light availability and neighborhood competition may favor species with contrasting leaf traits. | (Ackerly and Cornwell, 2007; Finegan et al., 2015) |
| Structural diversity  →  Net woody productivity | Association between horizontal structural heterogeneity and net woody productivity. | Neighborhood configuration may influence crown packing and light interception, with consequences for biomass accumulation. | (Deng et al., 2025; Zhai et al., 2024) |
| Phylogenetic diversity  →  Functional diversity | Covariation between taxonomic breadth and functional dissimilarity. | Phylogenetic niche conservatism predicts a relationship between relatedness and trait similarity; the taxonomy-derived tree provides an approximate representation of relatedness. | (Cavender-Bares et al., 2009; Srivastava et al., 2012) |
| Phylogenetic diversity  →  Net woody productivity | Association between taxonomic breadth and net woody productivity. | Taxonomically distinct species may differ in ecological strategies relevant to resource use and biomass accumulation. | (Cadotte et al., 2008; Paquette et al., 2015) |
| Functional diversity  →  Net woody productivity | Association between abundance-weighted functional dissimilarity and net woody productivity. | Trait differences may allow species to partition light, water, and nutrients, providing a potential basis for resource complementarity. | (Loreau and Hector, 2001; Tilman et al., 1997) |
| Functional traits  →  Net woody productivity | Association between community-weighted leaf trait composition and net woody productivity. | The mass-ratio hypothesis links ecosystem functioning to the traits of dominant species. Here, CWM values characterize trait composition weighted by relative stem abundance. | (Díaz et al., 2007; Finegan et al., 2015; Grime, 1998) |
| Species diversity  →  Structural diversity  →  Net woody productivity | Indirect association through horizontal structural heterogeneity. | Species differences may contribute to neighborhood heterogeneity associated with light interception and biomass accumulation. | (Deng et al., 2025; Forrester and Bauhus, 2016) |
| Species diversity →  Functional traits  →  Net woody productivity | Indirect association through community-weighted leaf trait composition. | Variation in species composition and relative abundance may shift CWM leaf traits associated with net woody productivity. | (Finegan et al., 2015; Grime, 1998) |
| Species diversity  →  Functional diversity  →  Net woody productivity | Indirect association through abundance-weighted functional dissimilarity. | Changes in species composition and relative abundance may alter functional dissimilarity and opportunities for resource partitioning. | (Laliberté and Legendre, 2010; Petchey and Gaston, 2006) |

**Note:** Species diversity, structural diversity, phylogenetic diversity, and functional diversity were represented by the Simpson index, DFSh, Faith-PD, and RaoQ, respectively. Functional traits were represented by PC1 derived from standardized CWM-LT, CWM-SLA, and CWM-LA; higher PC1 scores indicated higher CWM-SLA and CWM-LA but lower CWM-LT. Faith-PD was calculated from the taxonomy-derived proxy phylogeny. The proposed mechanisms provide ecological rationale for the pathways; path coefficients describe conditional associations and do not establish causality. The indirect pathways listed are illustrative, whereas indirect associations were calculated across all directed paths linking each predictor to net woody productivity in the fitted model.

### S2 Supplementary Results

#### S2.1 Univariate associations between community attributes and net woody productivity

Figure S1 Significant univariate associations between net woody productivity and community attributes

Note: (a) Community; (b) trees; (c) subtrees; and (d) woody lianas. Points represent 20 × 20 m subplots, and solid lines represent fitted linear relationships. Community attributes are shown on a standardized scale. Only associations with *p* < 0.05 are displayed; no significant associations were detected for shrubs. Trait abbreviations are defined in Section S1.1 and other indices in Table S3.

Univariate linear regressions examined associations between individual community attributes and net woody productivity (NWP). Community NWP was positively associated with CWM-LT and CWM-LDMC and negatively associated with CWM-SLA and CWM-LA (Figure S1a). Associations with CWM-LT and CWM-SLA were significant at p < 0.001, whereas those with CWM-LDMC and CWM-LA were significant at p < 0.05. Tree-stratum NWP was positively associated with CWM-LT, SDN, DFSv, and DFS and negatively associated with CWM-SLA (Figure S1b). Subtree-stratum NWP was positively associated with CWM-LDMC and negatively associated with CWM-LA, CWM-SLA, DFSv, and DFS (Figure S1c). No significant univariate associations were detected for shrub-stratum NWP. Significant associations for woody-liana NWP involved species, structural, functional, and phylogenetic diversity and CWM traits (Figure S1d).

#### S2.2 AIC-based multivariable regression models

The AIC-selected models explained 34.0% of the variation in community NWP, 22.6% in tree NWP, 21.5% in subtree NWP, 7.0% in shrub NWP, and 37.8% in woody liana NWP (Table 2). In both the community and tree-stratum models, CWM-LT and Simpson diversity had significant positive coefficients, while Pielou evenness and RaoQ had significant negative coefficients (Table S5).

Table S5 Standardized coefficients of retained variables in AIC-based stepwise regression models for net woody productivity

| **Response variable** | **Predictor** | **Dimension** | **Standardized β** | ***p*** |
| --- | --- | --- | --- | --- |
| Community NWP | Pielou | Species diversity | −0.743 | *p* < 0.01 |
|  | Simpson | Species diversity | 0.661 | *p* < 0.01 |
|  | Margalef | Species diversity | −0.660 | *p* < 0.05 |
|  | SDNS | Structural diversity | 0.651 | *p* < 0.01 |
|  | CWM-LT | Functional trait composition | 0.608 | *p* < 0.001 |
|  | RaoQ | Functional diversity | −0.459 | *p* < 0.001 |
|  | Faith-PD | Phylogenetic diversity | 0.450 | *p* ≥ 0.05 |
|  | DFSh | Structural diversity | 0.206 | *p* < 0.05 |
| Tree-stratum NWP | Simpson | Species diversity | 0.890 | *p* < 0.001 |
|  | Pielou | Species diversity | −0.585 | *p* < 0.01 |
|  | CWM-LT | Functional trait composition | 0.532 | *p* < 0.001 |
|  | RaoQ | Functional diversity | −0.312 | *p* < 0.01 |
|  | DFS | Structural diversity | 0.297 | *p* < 0.001 |
| Subtree-stratum NWP | DFSv | Structural diversity | −1.074 | *p* < 0.001 |
|  | DFS | Structural diversity | 0.760 | *p* < 0.01 |
|  | CWM-LDMC | Functional trait composition | 0.343 | *p* < 0.001 |
|  | CWM-WD | Functional trait composition | −0.182 | *p* < 0.05 |
| Shrub-stratum NWP | CWM-TDMC | Functional trait composition | −0.182 | *p* ≥ 0.05 |
|  | DFSv | Structural diversity | −0.169 | *p* < 0.05 |
|  | CWM-SLA | Functional trait composition | 0.126 | *p* ≥ 0.05 |
|  | MNTD | Phylogenetic diversity | 0.124 | *p* ≥ 0.05 |
| Woody liana NWP | Shannon | Species diversity | 0.854 | *p* < 0.01 |
|  | Faith-PD | Phylogenetic diversity | 0.633 | *p* < 0.01 |
|  | Margalef | Species diversity | −0.581 | *p* < 0.05 |
|  | SDNS | Structural diversity | −0.500 | *p* < 0.05 |
|  | CWM-LDMC | Functional trait composition | −0.490 | *p* < 0.001 |
|  | CWM-SLA | Functional trait composition | −0.282 | *p* < 0.05 |
|  | CWM-TDMC | Functional trait composition | −0.196 | *p* < 0.05 |
|  | CWM-WD | Functional trait composition | 0.179 | *p* < 0.05 |

Note: Values are standardized regression coefficients for predictors retained in the AIC-selected multiple-regression models. Positive and negative β values indicate conditional associations after accounting for the other predictors retained in the same model. Nonsignificant retained terms are included because AIC-based model selection did not require every coefficient to meet a significance threshold. NWP, net woody productivity; trait abbreviations are defined in Section S1.1, and other indices are defined in Table S3. Ordinary model-level R^2^ values and overall model tests are reported in main-text Table 2.

#### S2.3 PCA and multicollinearity diagnostics

The first principal component (PC1) explained 61.50% of the total variation in standardized CWM-LT, CWM-SLA, and CWM-LA (Table S6). CWM-SLA and CWM-LA had positive loadings on PC1, whereas CWM-LT had a negative loading. Higher PC1 scores therefore represented communities with higher CWM-SLA and CWM-LA but lower CWM-LT.

Table S6 PCA loadings of the CWM leaf traits used to derive PC1

| **Trait** | **PC1 loading** | **Ecological interpretation** |
| --- | --- | --- |
| CWM-LT | −0.438 | Leaf thickness and tissue construction |
| CWM-SLA | 0.707 | Leaf area per unit dry mass |
| CWM-LA | 0.556 | Leaf size and light capture potential |
| Explained variance | 61.50% | Proportion of trait variation explained by PC1 |

Note: PC1 was used as the leaf economics-related trait axis in the piecewise SEM. Higher scores corresponded to higher CWM-SLA and CWM-LA but lower CWM-LT.

Pairwise Pearson correlations and variance inflation factors were examined among the five explanatory variables included in the piecewise SEM. All absolute pairwise correlations were below 0.70 (Figure S2), and VIF values ranged from 1.290 to 2.875, remaining below the threshold of 3 (Table S7).

Figure S2 Pearson correlation matrix among variables included in the SEM

**Note:** Values and colors in the lower triangle indicate Pearson correlation coefficients; the upper triangle indicates significance. One, two, three, and four asterisks denote p < 0.05, 0.01, 0.001, and 0.0001, respectively. PC1 represents the leaf trait axis defined in Table S6; other abbreviations are defined in Table S3.

Table S7 Descriptive statistics and VIF values for variables included in the SEM

| **Community attribute** | **Variable** | **Mean ± SD** | **VIF** |
| --- | --- | --- | --- |
| Species diversity | Simpson | 0.88±0.08 | 2.875 |
| Structural diversity | DFSh | 0.65±0.04 | 1.447 |
| Phylogenetic diversity | Faith-PD | 83.67±15.73 | 1.568 |
| Functional diversity | RaoQ | 2.15±0.41 | 1.290 |
| Functional trait composition | PC1 | 0.00±1.36 | 1.519 |

**Note:** SD, standard deviation; VIF, variance inflation factor. Descriptive statistics refer to the unstandardized diversity indices and the derived PC1 scores. VIFs refer to the five variables assessed before SEM fitting.

#### S2.4 Structural equation model

The NWP component model of the piecewise SEM explained 19.1% of the variation in community NWP (R^2^ = 0.191). The model showed no significant lack of fit (χ^2^ = 2.846, df = 4, p = 0.584; Table S8). Species diversity and structural diversity, represented by Simpson and DFSh, respectively, had significant positive direct associations with community NWP, whereas the leaf economics-related trait axis PC1 had a significant negative direct association. Direct associations of Faith-PD and RaoQ with community NWP were not significant (Figure 4a). The positive direct association of species diversity with community NWP was partly offset by negative estimated indirect associations, primarily through PC1 (Figure 4b).

Table S8. Global model adequacy and explanatory power of the piecewise SEM

| **Category** | **Indicator** | **Result** | **Reference criterion / note** |
| --- | --- | --- | --- |
| Model goodness-of-fit | Log-likelihood-based generalized χ^2^ | 2.846 | — |
|  | Degrees of freedom (df) | 4 | — |
|  | Generalized chi-square test *p* value | 0.584 | No significant lack of fit |
| Component-model R^2^ | Structural diversity: DFSh | 0.294 | Variation explained by species diversity |
|  | Phylogenetic diversity: Faith-PD | 0.356 | Variation explained by species diversity |
|  | Functional trait composition: PC1 | 0.339 | Variation explained by species and structural diversity |
|  | Functional diversity: RaoQ | 0.220 | Variation explained by species and phylogenetic diversity |
|  | Community NWP | 0.191 | Variation explained by the five community attributes |

Note: Species diversity, structural diversity, phylogenetic diversity and functional diversity were represented by the Simpson index, DFSh, Faith-PD and RaoQ, respectively. Functional traits were represented by the first principal component derived from CWM-LT, CWM-SLA and CWM-LA. R^2^ values describe the variation explained by each component model. Model fit and path coefficients do not establish causal relationships.

#### S2.5 Spatial autocorrelation diagnostics

No significant global spatial autocorrelation was detected for community NWP (Moran’s *I* = 0.077, *p* = 0.161) or for residuals from the final community multiple-regression model (Moran’s *I* = 0.095, *p* = 0.092) under the specified spatial weights (Table S9).

Table S9. Global Moran’s I tests for community net woody productivity and final multiple-regression residuals

| **Analysis** | **Variable** | **N** | **Spatial weights** | **Moran’s I** | ***p* value** | **Conclusion** |
| --- | --- | --- | --- | --- | --- | --- |
| Global Moran’s I | Community NWP | 150 | Rook contiguity; row-standardized | 0.077 | 0.161 | No significant global spatial autocorrelation |
|  | Residuals from the final community-level regression model | 150 | Rook contiguity; row-standardized | 0.095 | 0.092 | No significant global residual spatial autocorrelation |

Note: The 150 contiguous 20 m × 20 m subplots were treated as neighbors only when they shared a 20 m edge. Significance was assessed using 99,999 unrestricted two-sided random permutations.

S3 Supplementary References

Ackerly, D. D., and Cornwell, W. K. (2007). A trait-based approach to community assembly: partitioning of species trait values into within- and among-community components. Ecol. Lett. 10, 135–145. doi: 10.1111/j.1461-0248.2006.01006.x

Buongiorno, J., Dahir, S., Lu, H.-C., and Lin, C.-R. (1994). Tree size diversity and economic returns in uneven-aged forest stands. For. Sci. 40, 83–103. doi: 10.1093/forestscience/40.1.83

Cadotte, M. W., Cardinale, B. J., and Oakley, T. H. (2008). Evolutionary history and the effect of biodiversity on plant productivity. Proc. Natl. Acad. Sci. U.S.A. 105, 17012–17017. doi: 10.1073/pnas.0805962105

Cavender-Bares, J., Kozak, K. H., Fine, P. V. A., and Kembel, S. W. (2009). The merging of community ecology and phylogenetic biology. Ecol. Lett. 12, 693–715. doi: 10.1111/j.1461-0248.2009.01314.x

Chave, J., Coomes, D., Jansen, S., Lewis, S. L., Swenson, N. G., and Zanne, A. E. (2009). Towards a worldwide wood economics spectrum. Ecol. Lett. 12, 351–366. doi: 10.1111/j.1461-0248.2009.01285.x

Clark, D. A., Brown, S., Kicklighter, D. W., Chambers, J. Q., Thomlinson, J. R., and Ni, J. (2001). Measuring net primary production in forests: concepts and field methods. Ecol. Appl. 11, 356–370. doi: 10.1890/1051-0761(2001)011[0356:MNPPIF]2.0.CO;2

Cornelissen, J. H. C., Lavorel, S., Garnier, E., Díaz, S., Buchmann, N., Gurvich, D. E., et al. (2003). A handbook of protocols for standardised and easy measurement of plant functional traits worldwide. Aust. J. Bot. 51, 335–380. doi: 10.1071/BT02124

Deng, X., Schmid, B., Bruelheide, H., Chen, C., Li, Y., Li, S., et al. (2025). Forest biodiversity increases productivity via complementarity from greater canopy structural complexity. Proc. Natl. Acad. Sci. U.S.A. 122: e2506750122. doi: 10.1073/pnas.2506750122

Díaz, S., Lavorel, S., de Bello, F., Quétier, F., Grigulis, K., and Robson, T. M. (2007). Incorporating plant functional diversity effects in ecosystem service assessments. Proc. Natl. Acad. Sci. U.S.A. 104, 20684–20689. doi: 10.1073/pnas.0704716104

Díaz, S., Kattge, J., Cornelissen, J. H. C., Wright, I. J., Lavorel, S., Dray, S., et al. (2016). The global spectrum of plant form and function. Nature 529, 167–171. doi: 10.1038/nature16489

Dong, L., Bettinger, P., and Liu, Z. (2024). Stand spatial structural diversity: developing and validating a novel index. For. Ecol. Manage. 569: 122157. doi: 10.1016/j.foreco.2024.122157

Ehbrecht, M., Seidel, D., Annighöfer, P., Kreft, H., Köhler, M., Zemp, D. C., et al. (2021). Global patterns and climatic controls of forest structural complexity. Nat. Commun. 12: 519. doi: 10.1038/s41467-020-20767-z

Faith, D. P. (1992). Conservation evaluation and phylogenetic diversity. Biol. Conserv. 61, 1–10. doi: 10.1016/0006-3207(92)91201-3

Finegan, B., Peña-Claros, M., de Oliveira, A., Ascarrunz, N., Bret-Harte, M. S., Carreño-Rocabado, G., et al. (2015). Does functional trait diversity predict above-ground biomass and productivity of tropical forests? Testing three alternative hypotheses. J. Ecol. 103, 191–201. doi: 10.1111/1365-2745.12346

Forrester, D. I., and Bauhus, J. (2016). A review of processes behind diversity–productivity relationships in forests. Curr. For. Rep. 2, 45–61. doi: 10.1007/s40725-016-0031-2

Grime, J. P. (1998). Benefits of plant diversity to ecosystems: immediate, filter and founder effects. J. Ecol. 86, 902–910. doi: 10.1046/j.1365-2745.1998.00306.x

Hui, G., Zhao, Z., Hu, Y., Zhang, G., Zhang, G., Chen, S., et al. (2023). Research on the measurement method of forest spatial structure diversity based on 4 neighborhood tree relationship. J. Beijing For. Univ. 45, 18–26. doi: 10.12171/j.1000-1522.20220282

Laliberté, E., and Legendre, P. (2010). A distance-based framework for measuring functional diversity from multiple traits. Ecology 91, 299–305. doi: 10.1890/08-2244.1

Lei, X. D., Tang, M. P., Lu, Y. C., Hong, L. X., and Tian, D. L. (2009). Forest inventory in China: status and challenges. Int. For. Rev. 11, 52–63. doi: 10.1505/ifor.11.1.52

Liang, J., Crowther, T. W., Picard, N., Wiser, S., Zhou, M., Alberti, G., et al. (2016). Positive biodiversity-productivity relationship predominant in global forests. Science 354: aaf8957. doi: 10.1126/science.aaf8957

Loreau, M., and Hector, A. (2001). Partitioning selection and complementarity in biodiversity experiments. Nature 412, 72–76. doi: 10.1038/35083573

Malhi, Y., Baker, T. R., Phillips, O. L., Almeida, S., Alvarez, E., Arroyo, L., et al. (2004). The above-ground coarse wood productivity of 104 Neotropical forest plots. Glob. Change Biol. 10, 563–591. doi: 10.1111/j.1529-8817.2003.00778.x

Paquette, A., Joly, S., and Messier, C. (2015). Explaining forest productivity using tree functional traits and phylogenetic information: two sides of the same coin over evolutionary scale? Ecol. Evol. 5, 1774–1783. doi: 10.1002/ece3.1456

Pérez-Harguindeguy, N., Díaz, S., Garnier, E., Lavorel, S., Poorter, H., Jaureguiberry, P., et al. (2013). New handbook for standardised measurement of plant functional traits worldwide. Aust. J. Bot. 61, 167–234. doi: 10.1071/BT12225

Perles-Garcia, M. D., Kunz, M., Fichtner, A., Härdtle, W., and von Oheimb, G. (2021). Tree species richness promotes an early increase of stand structural complexity in young subtropical plantations. J. Appl. Ecol. 58, 2305–2314. doi: 10.1111/1365-2664.13973

Petchey, O. L., and Gaston, K. J. (2006). Functional diversity: back to basics and looking forward. Ecol. Lett. 9, 741–758. doi: 10.1111/j.1461-0248.2006.00924.x

Pielou, E. C. (1966). The measurement of diversity in different types of biological collections. J. Theor. Biol. 13, 131–144. doi: 10.1016/0022-5193(66)90013-0

Rao, C. R. (1982). Diversity and dissimilarity coefficients: a unified approach. Theor. Popul. Biol. 21, 24–43. doi: 10.1016/0040-5809(82)90004-1

Shannon, C. E. (1948). A mathematical theory of communication. Bell Syst. Tech. J. 27, 379–423. doi: 10.1002/j.1538-7305.1948.tb01338.x

Simpson, E. H. (1949). Measurement of diversity. Nature 163, 688. doi: 10.1038/163688a0

Srivastava, D. S., Cadotte, M. W., MacDonald, A. A. M., Marushia, R. G., and Mirotchnick, N. (2012). Phylogenetic diversity and the functioning of ecosystems. Ecol. Lett. 15, 637–648. doi: 10.1111/j.1461-0248.2012.01795.x

Tilman, D., Knops, J., Wedin, D., Reich, P., Ritchie, M., and Siemann, E. (1997). The influence of functional diversity and composition on ecosystem processes. Science 277, 1300–1302. doi: 10.1126/science.277.5330.1300

Ulanowicz, R. E. (2001). Information theory in ecology. Comput. Chem. 25, 393–399. doi: 10.1016/S0097-8485(01)00073-0

Villéger, S., Mason, N. W. H., and Mouillot, D. (2008). New multidimensional functional diversity indices for a multifaceted framework in functional ecology. Ecology 89, 2290–2301. doi: 10.1890/07-1206.1

Webb, C. O., Ackerly, D. D., McPeek, M. A., and Donoghue, M. J. (2002). Phylogenies and community ecology. Annu. Rev. Ecol. Syst. 33, 475–505. doi: 10.1146/annurev.ecolsys.33.010802.150448

Wright, I. J., Reich, P. B., Westoby, M., Ackerly, D. D., Baruch, Z., Bongers, F., et al. (2004). The worldwide leaf economics spectrum. Nature 428, 821–827. doi: 10.1038/nature02403

Wu, J., Zhu, J., Ai, X., Yao, L., Guo, Q., Xue, W., et al. (2022). A dataset of woody plant biomass models for subtropical mixed evergreen and deciduous broad-leaved forests. China Scientific Data 7, 342–353. doi: 10.11922/11-6035.csd.2022.0037.zh

Wu, J., Xiang, Q., Xia, Y., Lei, J., Ai, X., Wang, Z., et al. (2025). Effects of stand structural changes on vegetation carbon sequestration capacity during secondary succession in subtropical evergreen-deciduous broadleaved mixed forests in central China. J. Environ. Manage. 396: 128016. doi: 10.1016/j.jenvman.2025.128016

Zhai, L., Will, R. E., and Zhang, B. (2024). Structural diversity is better associated with forest productivity than species or functional diversity. Ecology 105: e4269. doi: 10.1002/ecy.4269
